# Obtaining Cross-Sections of Paint Layers in Cultural Artifacts Using Femtosecond Pulsed Lasers

**DOI:** 10.3390/ma10020107

**Published:** 2017-01-26

**Authors:** Takaaki Harada, Stephanie Spence, Athanasios Margiolakis, Skylar Deckoff-Jones, Rebecca Ploeger, Aaron N. Shugar, James F. Hamm, Keshav M. Dani, Anya R. Dani

**Affiliations:** 1 Femtosecond Spectroscopy Unit, Okinawa Institute of Science and Technology Graduate University, 1919-1 Tancha, Onna-son, Kunigami, Okinawa 904-0495, Japan; takaaki.harada@oist.jp (T.H.); mthanasis@gmail.com (A.M.); sdeckoff@tulane.edu (S.D.-J.); kmdani@oist.jp (K.M.D.); 2 Art Conservation Program, Science and Technology Group, Okinawa Institute of Science and Technology Graduate University, 1919-1 Tancha, Onna-son, Kunigami, Okinawa 904-0495, Japan; shspence@gmail.com; 3 Art Conservation Program, Buffalo State College, State University of New York, Buffalo, NY 14222, USA; ploeger@buffalostate.edu (R.P.); shugaran@buffalostate.edu (A.N.S.); hammjf@buffalostate.edu (J.F.H.)

**Keywords:** ultrafast laser, art conservation, material processing, CW laser, cross-section analysis

## Abstract

Recently, ultrafast lasers exhibiting high peak powers and extremely short pulse durations have created a new paradigm in materials processing. The precision and minimal thermal damage provided by ultrafast lasers in the machining of metals and dielectrics also suggests a novel application in obtaining precise cross-sections of fragile, combustible paint layers in artwork and cultural heritage property. Cross-sections of paint and other decorative layers on artwork provide critical information into its history and authenticity. However, the current methodology which uses a scalpel to obtain a cross-section can cause further damage, including crumbling, delamination, and paint compression. Here, we demonstrate the ability to make controlled cross-sections of paint layers with a femtosecond pulsed laser, with minimal damage to the surrounding artwork. The femtosecond laser cutting overcomes challenges such as fragile paint disintegrating under scalpel pressure, or oxidation by the continuous-wave (CW) laser. Variations in laser power and translational speed of the laser while cutting exhibit different benefits for cross-section sampling. The use of femtosecond lasers in studying artwork also presents new possibilities in analyzing, sampling, and cleaning of artwork with minimal destructive effects.

## 1. Introduction

Over the past few decades, ultrashort pulsed lasers have introduced new methods to material processing and machining [1]. Due to extremely short femtosecond scale pulse durations at the focal spot of the laser, ultrashort pulses provide extremely high peak optical intensities [2], which drive strong nonlinearities and can result in laser-induced optical breakdown of the material. Thus, one can alter the local structure of the material or even create a void with high spatial precision. This has led to a number of useful applications of ultrafast laser processing, such as high-precision micromachining [1,3,4], laser-processed black silicon for photovoltaic applications [5], and engineering opto-electronic properties through femtosecond laser ablation for terahertz devices [6]. Another important advantage of femtosecond laser processing is the minimization of thermal damage [3,4]. In contrast to continuous-wave (CW) lasers, the short pulse duration in ultrafast lasers is typically smaller than electron-phonon interaction times, thus minimizing any heat diffusion outside the focal area of the laser [3,4]. Therefore, one can restrict the laser-induced changes to the focal spot, while minimizing thermal damage to the surrounding material [3,4]. The high precision, reduction of damage to surrounding material, and the minimization of thermal effects suggests another novel application of femtosecond laser processing—cutting through fragile paint layers in valuable artwork and cultural artifacts to obtain cross-sections.

Cross-section analysis of artwork is a conventional way to investigate the layered structure of paint, identify pigments, binding mediums and varnishes, and restoration materials from prior treatment campaigns. Commonly, small samples of decorative paint layers are removed by using a scalpel, applying lateral pressure with a scalpel to obtain a piece of paint along tacking margins or from an existing area of loss. The small piece is typically embedded in an epoxy resin and polished with fine micromesh to expose the cross-section of the paint layers. However, in friable paintings, the act of removing a sample using a scalpel may cause substantial damage to the surrounding paint or the small removed fragment, lowering its integrity. To avoid the potential damage to paint layers due to scalpel pressure, laser processing is a potential alternative. In art conservation, CW, μs-pulsed, and ns-pulsed lasers such as Q-switched Nd:YAG [7,8] or Er:YAG lasers [9,10], with wavelengths typically in the near IR, visible, and near UV ranges, have become popular as a cleaning method for a wide range of materials such as marble sculptures, historic building facades, metals, and paintings. Using a KrF excimer laser, Teule et al. demonstrated the topological removal of the varnish layer with a continuous-wave laser to reveal the paint layer underneath [11]. The use of CW lasers to burn away unwanted residues on top of artwork is partly possible by using wavelength selectivity to maximize absorption in the layers to be removed, and minimize any effects on the underlying artwork. However, in the process of taking cross-sections of the artwork itself, such wavelength selectivity is not an option, and CW lasers risk oxidative damage to the surrounding areas, though they may overcome some of the challenges afforded by a scalpel treatment. In contrast, femtosecond pulsed lasers offer the possibility of cutting cross-sections of paint layers with minimal damage to surrounding artwork due to its inherent reliance on high peak power and non-thermal effects to cut through a material. Previously, the use of femtosecond lasers in art conservation has been explored by studying the possibility of removal of varnish from paint using UV pulses [12], as well as to non-destructively study pigments and compositions of artworks through femtosecond pump-probe spectroscopy [13]. However, no studies have been performed to-date to study the appropriateness and potential utility of femtosecond lasers to obtain cross-sections of paint layers.

In this article, we demonstrate the ability to take cross-sections of a range of paint types using a femtosecond pulsed laser to cut through paint layers with limited to no damage to the surrounding artworks. We use fragile pieces of oil paints ranging from the 19th century to the 1980s that have been shown to easily disintegrate under scalpel pressure. We also show that femtosecond pulsed lasers avoid the oxidation damage that CW lasers cause in the same paint layers. The precision of the femtosecond pulsed laser cut is due to the formation of a plasma in the tightly focused laser spot size. We investigate the cross-sections obtained as a function of the laser power and cutting speed to experimentally identify the advantages and dis-advantages associated with the different laser processing conditions. We show that any oxidized material deposited as a result of the femtosecond laser processing along the vertical walls of the cut can be easily removed with mineral spirits. In general, our work shows that the femtosecond laser processing of fragile paint layers opens new possibilities in analyzing, sampling, and cleaning fragile valuable artwork and cultural property with minimal destructive effects.

## 2. Sample Description and Experimental Setup

Paint samples **1**–**5** were obtained from the study collection in the Art Conservation Department at Buffalo State, State University of New York, as summarized in Table 1. Sample **1** is a segment of an oil painting, c. 19th century, with a natural resin varnish. The painting section is mostly brown oil paint applied on top of a lead white ground and canvas substrate. The paint and ground have a total thickness of ~200 μm. Sample **2** is a segment of a painting entitled “Still Life with Green Pitcher” by Christopher Kressy, produced in 1977. The turquoise oil paint was applied over a white ground on a linen canvas. The total thickness of the paint was ~250 μm. The white ground was also applied to the verso of the canvas, resulting in the thickness of ~740 μm. This technique helps strengthen the canvas to minimize cracking that can occur from stress over time. Samples **3**–**5** are different segments of a painting entitled “54 Rue de l’agé- 1912” by Arthur Page produced in the 1980s. Oil paints were applied on a pre-primed canvas which contained either ground or a “primer” layer. The thickness of the canvas with ground was ~370 μm. The paints and varnish layers were applied differently in each sample. Sample **3** contains a white oil paint with a thickness of ~220 μm, and varnish was applied beneath the top white paint layer(s). Sample **4** contains a red oil paint with a thickness of ~120 μm. Varnish was not applied to this sample segment. Sample **5** contains yellow and white oil paints with a thickness of ~100 μm. Varnish was applied to this sample segment. The paint layers of samples **3**–**5** are extremely friable, and easily flake off. Some areas of the painting also exhibit alligatoring that occurs as a result of the varied drying process. For convenience, in our current setup, about 1 cm^2^ segments of the samples were mounted on glass slides with tape for the present study. With a suitably modified apparatus, larger pieces of artwork (such as entire paintings) could be appropriately mounted for similar studies.

**Figure 1:**
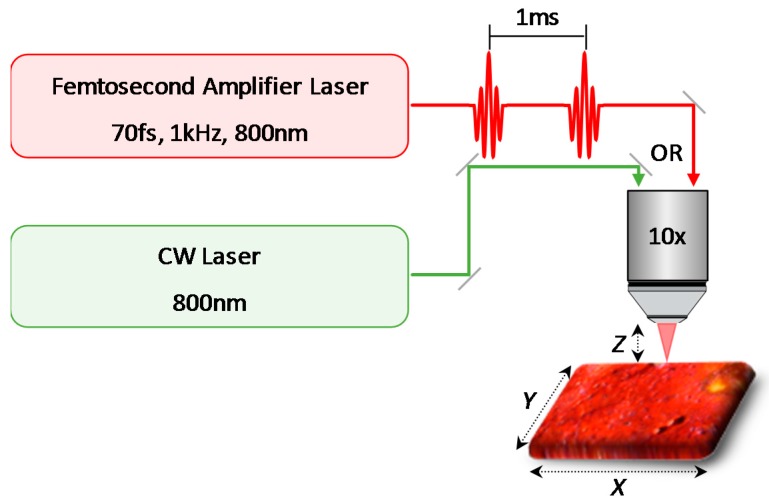
Schematic illustration of the laser cut processing of paints with either a femtosecond or continuous-wave (CW) laser source. The laser beams are focused at each sample through a 10× objective lens. The samples are mounted on a motorized XY translational stage with variable speeds. To completely cut through all the paint layers, the sample is vertically shifted along the Z direction by re-focusing after the completion of each trench.

Figure 1 is a schematic illustration of the experimental setup with either a femtosecond or CW laser source for the laser processing. The femtosecond laser system used in this study was a Ti:sapphire amplifier centered at 800 nm with a repetition rate of 1 kHz and a pulse duration of 70 fs. Briefly, seed pulses from a femtosecond oscillator were amplified by a high-power Q-switched Nd:YLF pump laser in a second Ti:sapphire amplifier cavity. Several cycles of the seed amplification produced a 1 kHz train of femtosecond pulses with up to 5 W of average power. A small part of the amplified femtosecond laser beam (a few mW) was guided to an optical microscope. The laser beam was focused at each sample through a 10× objective lens (NA 0.30), to give a spot size of ~20 μm, and the power at the sample position was set to 0.2, 2, and 20 mW, corresponding to a fluence (energy contained in each pulse per unit area) of 0.064, 0.640, and 6.4 J/cm^2^, respectively. The samples on the glass slide were mounted on a motorized computer-controlled two-dimensional translational stage attached to the microscope, which provides precisely controlled laser cutting with variable speeds of 0.1, 1, and 10 mm/s. After each pass along the X direction, the sample was moved horizontally in the Y direction by 1 μm. This procedure was typically repeated 50 times to create a trench of ~100 μm width. We estimate the depths of the trench to be about 3, 30, and 300 μm when the input powers were 0.2, 2, and 20 mW (0.064, 0.64, and 6.4 J/cm^2^), respectively. In order to completely cut through all the paint layers, the sample was vertically shifted along the Z direction after the completion of each trench. For ~250 μm depths required in our samples, a 100 μm trench width was easily achievable. Thinner trench widths may be achievable for this depth with a suitable numerical aperture for the microscope objective. Conversely, for thicker paint samples, wider trenches may be necessary due to constraints in the Rayleigh range and beam waist, which essentially determines the width:depth aspect ratio of a cut during femtosecond laser processing of materials. We note that although the sample was moved relative to the laser beam in our setup, a suitable modification can easily allow for a stationary sample while the laser beam is moved. For example, as in the case of various two-photon microscope configurations, the actual painting could be held fixed, while the optical spot size is translated via automated galvanometer mirrors. Alternately, recent developments in femtosecond fiber lasers could also potentially ease the experimental configurations for actual artwork due to their portable size and the convenience associated with fiber delivery of light.

In order to compare the effect of femtosecond pulsed lasers with CW lasers, the output of a CW Ti:sapphire laser with the same center wavelength of 800 nm was guided through the same setup. Its power at the sample position was set to 60 mW or less, and the XY translational speed was 10 mm/s. The samples were cleaned with a standard mineral spirit. In order to understand the effects of the femtosecond laser processing, optical images of the laser cut were taken with another microscope with fiber lighting in top view, as well as cross-sectional view. Additionally, in order to better view the layered structure of the paint, higher resolution scanning electron microscopy (SEM) images were also taken of the cross-sections. This also allowed the assessment of the cuts at different angles. We note that the SEM images of the cross-sections were taken without applying standard polishing procedures to the cross-section. With the SEM, samples were viewed in both backscattered and secondary electron modes using 1.00 kV and 5.00 kV. As necessary, some samples were coated with carbon in order to decrease charging and improve the image.

## 3. Results and Discussion

### 3.1. Comparison of Scalpel, CW Laser, and Femtosecond Pulsed Laser

**Figure 2:**
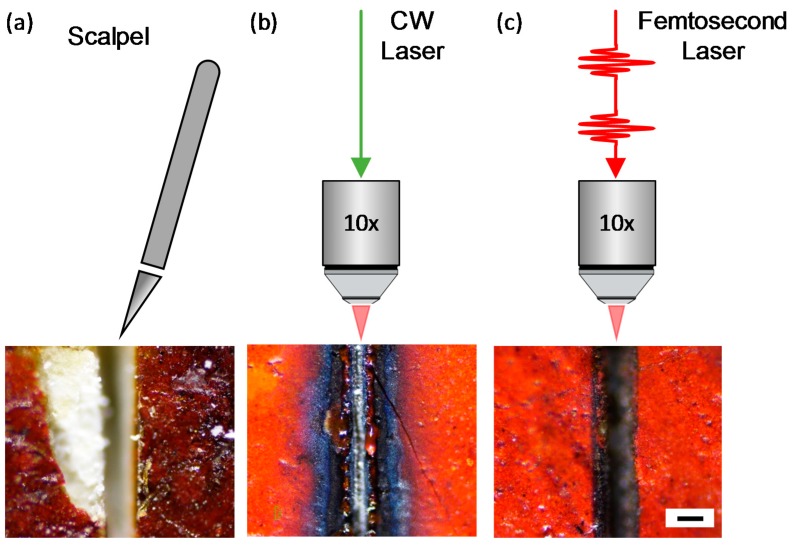
Schematic illustrations and optical images of the paint sample **1** which was cut by a (**a**) scalpel; (**b**) 60-mW CW laser; and (**c**) 2-mW (0.64-J/cm^2^) femtosecond laser. The laser beams were focused onto the sample through a 10× objective lens. The black scale bar is 100 μm.

Figure 2 shows a comparison of cutting methodologies to obtain pieces of paint samples for cross-section analysis. The conventional sampling with a scalpel results in crumbling of the paint layers in sample **1** (Figure 2a). A small piece of the top paint layer crumbled off, revealing the ground layer and canvas, and cracks were observed in the surrounding regions of the original paint. Next, to study the effect of the CW laser, the output of the CW Ti:Sapphire laser was focused onto sample **1** through the optical microscope. The paint samples shown in Figure 2a,b may show different colors, but they are the same paint in proximity. For low-power (up to 20 mW or 6.4 J/cm^2^), the CW laser was unable to cut through the paint layer. At significantly higher powers (~60 mW), the CW laser begins to leave substantial darkening along the laser beam path. We also note that there is significant modification of the region around the focal point of the laser, extending to several hundred microns (Figure 2b). We expect that exposure of the CW laser generates heat at the focal point, and its accumulation causes significant oxidation and modification on the sample surface [14,15,16]. In contrast, the femtosecond laser demonstrates a clean cut and minimal darkening on the sample (Figure 2c). The resultant cut, taken at 2 mW (0.64 J/cm^2^)—much lower average power than the CW laser at the sample—and 10-mm/s translational speed of the XY stage shows insignificant surface modification in the region beyond the cut. The estimated laser spot size of ~20 μm and the intentional XY translational motion described in the experimental section result in a typical trench width of ~100 μm (Figure 2c). In principle, this can be reduced to few tens of micrometers, limited by the focal spot size of the laser beam. Although the average power of the femtosecond laser is 30 times less than that of the CW laser, the high peak power results in the formation of a plasma at the focal point due to multiphoton ionization [4,17], which removes the required paint material [1,3,4].

### 3.2. Comparison of the Effects of CW and Femtosecond Pulsed Lasers on a Variety of Paint Samples

**Figure 3:**
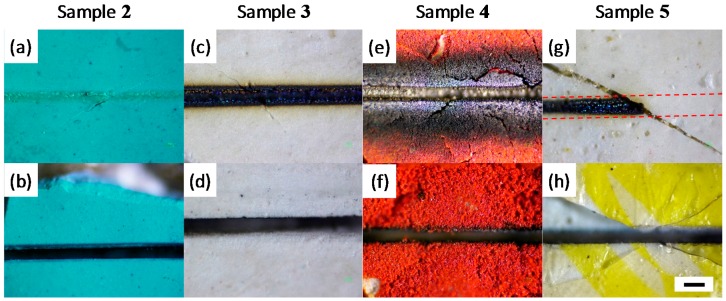
Optical images of trenches of samples **2**–**5** with the 60-mW CW laser (top row) and 2-mW (0.64-J/cm^2^) femtosecond laser (bottom row) for sample **2**—panels (**a**,**b**); sample **3**—panels (**c**,**d**); sample **4**—panels (**e**,**f**); and sample **5**—panels (**g**,**h**). The red dashed lines on panel (**g**) shows the CW beam path. None of the CW measurements resulted in a cut, even with 30 times higher average power. Note that the panels (**g**,**h**) show different color, but are originated from the same paint in proximity. The black scale bar is 100 μm.

**Figure A1:**
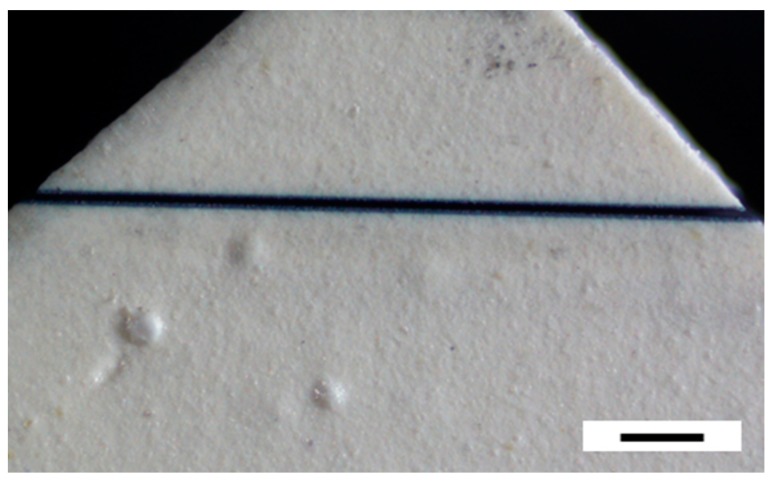
Optical image of a trench created with the femtosecond laser on a segment of alkyd paint sample **6**. The applied alkyd paint was produced by Winsor & Newton Griffin. The 220-μm paint was casted on a 90-μm polyester film in 2006. The total thickness of the paint was ~310 μm. The black scale bar is 200 μm. The 800-nm femtosecond laser cutting was performed at 2 mW with the XY translational speed of 1 mm/s.

Next, we compare the effects of the femtosecond laser with those of the CW laser on four other paint samples **2**–**5**, which are all friable and easily disintegrate under the application of a scalpel. In sample **2**, exposure to the 60-mW CW laser altered the top layer (as observed in an optical image after laser processing), but does not manage to cut through the paint layer (Figure 3a). In contrast, Figure 3b shows a clean cut produced by just 2 mW (0.64 J/cm^2^) of the femtosecond pulsed laser processing of sample **2**. Similar to the result in Figure 2c, despite the low average power, the high peak power of a femtosecond pulsed laser at the focal point creates a plasma and removes the paint layers with high precision and minimal thermal damage to surrounding paint. We note the presence of a thin layer of oxidized deposits after femtosecond laser processing just at the edges of the cut and along the vertical walls. This layer is easily removable with mineral spirits, and is discussed further below. In sample **3**, exposure to the 60-mW CW laser results in significant charring and darkening of the paint layers along the laser beam path (Figure 3c). Again, the CW laser is unable to cut through the paint layer, even at this high average power. In contrast, using the femtosecond pulsed laser on sample **3** results in a clean trench. In this sample, the appearance of some oxidized deposits on the surface post femtosecond laser processing is clearer. These deposits were also easily removed with mineral spirits, suggesting a superficial deposition. In sample **4**, Figure 3e,f also exhibits that the femtosecond laser processing results in a clean trench, while the CW laser shows significant oxidation, discoloration, and damage to surrounding regions. In Figure 3g,h, sample **5** shows different colors, but they are the same paint in proximity. In Figure 3g, the red dashed lines represent the CW laser path, and the diagonal crack is due to loss of the varnish layer. It is interesting to note that CW laser exposure causes charring in the regions of the sample unprotected by varnish, while the regions protected by the varnish layer are unaltered by the CW laser. In contrast, Figure 3h demonstrates that femtosecond laser processing results in a clean trench on sample **5** with or without the presence of varnish. In particular, the femtosecond laser cut on sample **5** did not cause further cracking of the varnish layer, as prepared with a scalpel. Aside from oil paints (samples **2**–**5**), femtosecond laser cutting is also applicable to other types of paints, including alkyd paints (Figure A1). In general, we observe that the effect of the CW laser varies significantly depending on the type of paint material, while the femtosecond laser produces a relatively uniform result, independent of the specific paint material. This is an important advantage when processing art materials, as one often encounters a wide range of art materials within a single artefact. 

### 3.3. Effects of Average Power and Cutting Speed

**Figure 4:**
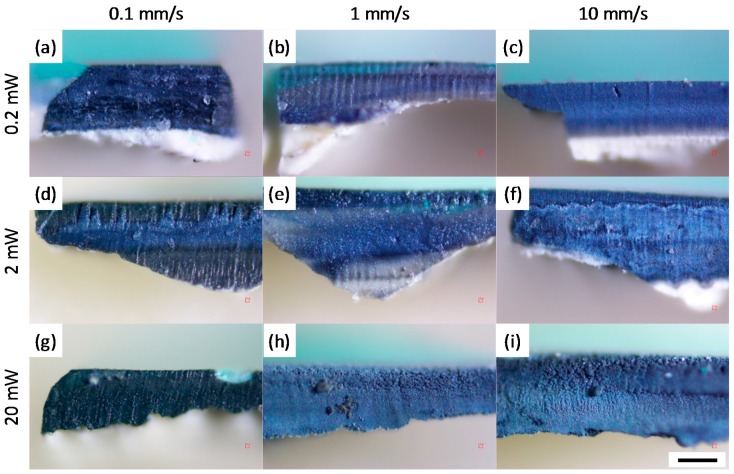
Optical images of sample **2** cross-sections showing effects of translational speeds (0.1, 1 and 10 mm/s—left, middle and right columns, respectively) and input power (0.2, 2 and 20 mW or 0.064, 0.64 and 6.4 J/cm^2^—top, center and bottom rows, respectively) after the femtosecond laser cutting. Inhomogeneous thicknesses of the paint layers are due to roughness of the applied linen canvas. The black scale bar is 100 μm. Cross-sections in panels (**a**–**c**) are obtained when changing translational speeds to 0.1, 1 and 10 mm/s, respectively, with a constant input power of 0.2 mW (0.064 J/cm^2^). Similarly, cross-sections in panels (**d**–**f**) are obtained under the same range of translational speeds of 0.1, 1 and 10 mm/s, respectively, but the input power is increased to 2 mW (0.64 J/cm^2^). Input power is further increased to 20 mW (6.4 J/cm^2^) to obtain cross-sections in panels (**g**–**i**) under the translational speeds of 0.1, 1 and 10 mm/s, respectively.

In order to understand the femtosecond laser cutting process of the paint layers, we next investigate the effects of the XY translational speed and input laser power on sample **2**. Cross-section images of the paint under various conditions were obtained under a light microscope (Figure 4). At all input powers, slower translational speed exhibited darkened cross-sections due to the previously-discussed deposition of oxidized material onto the cross-section. Slow translational speeds lead to increased exposure of each region of the paint layer to multiple laser pulses, resulting in larger ablated volumes, which creates more deposits. In contrast, faster speeds of the XY translational stage show significantly less deposits on the cross-sections. Similarly, darkening of the cross-sections due to oxidized deposits also increases with increasing laser power. In addition to the presence of oxidized deposits, the laser input power and XY translational speed also influence the practical question of the time it takes to make a cut through all the paint layers. For instance, the translational speed of 0.1 mm/s took at least 15 min to create a trench of 2 mm long in the sample, through the 250-μm paint layer with a 100-μm width at the input power of 20 mW (6.4 J/cm^2^). Similarly, for very low powers, we observed less effective laser cutting, which required multiple passes and a larger number of fine vertical steps of the sample to achieve the same depth of cut. Expectedly, at the higher input powers of 2 and 20 mW (0.64 and 6.4 J/cm^2^, respectively), the plasma removes paint material more effectively, thus requiring fewer passes. Overall, depending on the specific application, one can find a balance between speed and efficiency of the cut versus potential for more debris and oxidized deposits when varying the laser power and speed of the cut. 

**Figure 5:**
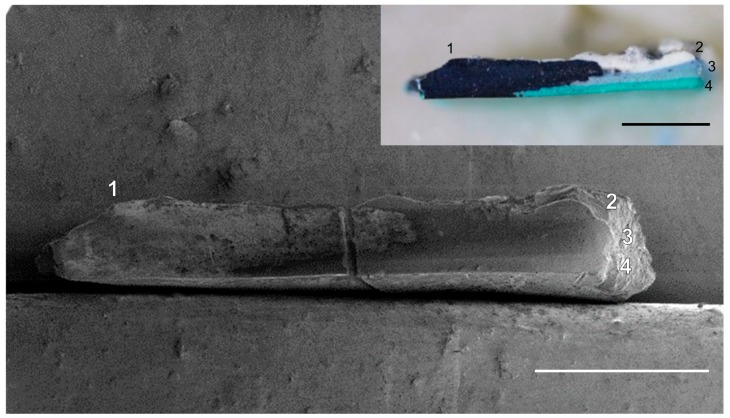
SEM image of cross-section of sample **2** obtained by the femtosecond laser cut at 20 mW (6.4 J/cm^2^) with the XY translational speed of 10 mm/s. The inset shows the optical image of the cross-section. The as-obtained topography of superficial oxidized deposits on the cross-section (**1**) is observable while the cleaned paint layers (**2**–**4**) show smooth cut. The scale bars are 500 μm.

Lastly, we show that the oxidized deposits on the cross-sectional surfaces are easily removable with mineral spirits, which is usually safe to apply to oil paint films. To demonstrate the removal of dark deposits, the cross-section of sample **2** shown in Figure 4i is partially cleaned. A secondary electron image of a cross-section of sample **2** reveals its cleaned and as-obtained topography (Figure 5). The superficial oxidized deposits on the cross-section are observable in both the SEM and optical images. The low contrast on the cleaned paint layers in the SEM image shows the smooth surface topography of the cross-section without polishing. The cleaned part of the cross-section also exhibits individual paint layers that are unobservable in the oxidized part. Further studies into the elemental composition of the oxidized deposit could reveal more about the mechanism of deposition, and potentially allow optimization of the femtosecond laser cutting process to minimize any associated damage to the artwork. Depositing a protective easily-removable layer prior to femtosecond laser processing in order to protect the artwork from the oxidized deposit is also a possibility to minimize damage. We note that the paint cross-section shown here has not been polished prior to imaging, as would have been necessary and standard for traditional scalpel cross-sections. The potential to eliminate the need for polishing can thus avoid problems during the polishing process, such as cross-contamination between paint layers, or with sand particles [18]. We also note that under a more stringent experimental geometry, it may be possible to employ laser induced breakdown spectroscopy (LIBS) while making the cut, in order to gain more information about the sample [19].

## 4. Conclusions

We present controlled cutting of cross-sections of fragile paint layers with femtosecond pulsed laser. Focusing of the high peak intensity and short pulse-duration of the femtosecond laser pulses creates a plasma at the focal point, which has the ability to cut through a range of fragile paint materials without the significant damage that scalpel pressure and heat accumulation by the CW laser would cause. Changing various parameters associated with femtosecond laser processing—such as the XY translational speed and input laser powers—allows one to optimize the speed, efficiency, and potential thermal damage to artwork while making the cut. Other parameters, such as the laser wavelength, pulse duration, and focal spot size could also be optimized based on the specific composition of the paint materials. Together, they provide a powerful toolbox to control the femtosecond laser cutting process, potentially extending its utility over a wider range of applications in studying and preserving cultural artefacts. Comparisons of the effects of femtosecond laser pulses to CW light at other wavelengths could also provide more insight into the utility of this technique to the field of art conservation. For example, femtosecond lasers could provide alternatives in laser cleaning, where CW lasers have the advantage of addressing unwanted layers through wavelength selectivity, but could nonetheless cause significant thermal damage to underlying artwork. Femtosecond lasers—which have far less wavelength selectivity—could potentially use spatial selectivity through tight focusing and potentially eliminate thermal damage. In general, the femtosecond laser processing of fragile art materials potentially presents new possibilities in analyzing, sampling, and cleaning artwork with minimal destructive effects.

## Figures and Tables

**Table 1:** Summary of oil paint samples **1**–**5**.

Paint	Color	Year	Paint Thickness (μm)	Total Thickness (μm)	Varnish
Sample **1**	Red/Brown ^1^	19th Century	-	200	Top
Sample **2**	Turquoise	1977	250	740	-
Sample **3**	While	1980s	220	590	Underneath
Sample **4**	Red	1980s	120	490	-
Sample **5**	Yellow-White ^1^	1980s	100	470	Top

^1^ The paint samples may show different colors but they are the same paint in proximity.
